# Use of kilovoltage X-ray volume imaging in patient dose calculation for head-and-neck and partial brain radiation therapy

**DOI:** 10.1186/1748-717X-5-29

**Published:** 2010-04-19

**Authors:** Weigang Hu, Jinsong Ye, Jiazhou Wang, Xuejun Ma, Zhen Zhang

**Affiliations:** 1 Department of Radiation Oncology, Cancer Hospital, Department of Oncology, Shanghai Medical college, Fudan University, Shanghai, China; 2 Department of Radiation Oncology, Swedish Cancer Institute, Seattle, WA, USA

## Abstract

**Background:**

To evaluate the accuracy of using kilovoltage x-ray cone-beam computed tomography (kV-CBCT) imaging for in vivo dose calculations.

**Methods:**

A Region-of-Interest (ROI) CT number mapping method was developed to generate the cone-beam CT number vs. relative electron density calibration curve for 3D dose calculations. The stability of the results was validated for three consecutive months. The method was evaluated on three brain tumors and three head-and-neck tumor cases. For each patient, kV-CBCT images were acquired on the first treatment day and two-week intervals on the Elekta XVI system. The delivered dose distributions were calculated by applying the patients' treatment plans to the kV-CBCT images. The resulting dose distributions and dose volume histograms (DVHs) of the tumor and critical structures were compared to the original treatment plan.

**Results:**

The kV-CBCT electron density calibration was stable within 1.5% over a three-month period. The DVH and dose distribution comparison based on the planning CT and the initial kV-CBCT showed good agreements for majority of cases. The doses calculated from the planning CT and kV-CBCT were compared on planes perpendicular to the beam axes and passing through the isocenter. Using γ analysis with a criterion of 2 mm/2% and a threshold of 10%, more than 99.5% of the points on the iso-planes exhibited γ <1. For one patient, kV-CBCT images detected 5.8% dose variation in the right parotid due to tumor shrinkage and patient weight loss.

**Conclusions:**

ROI mapping method is an effective method for the creation of kV-CBCT electron density calibration curves for head-and-neck and brain tumor patients. Dose variations as monitored using kV-CBCT imaging suggest that some patients can benefit from adaptive treatment plan re-optimization.

## Background

Patients with head-and-neck and definitive brain tumor are routinely treated with intensity-modulated radiotherapy (IMRT) to enable delivery of highly conformal dose distribution to the tumor while sparing surrounding critical structures. Precise target localization is important for such treatments [1-3]. Ideally, the cumulative dose delivered over the whole treatment course should match the total planned dose. However, many uncertainties can be incurred due to patient set-up, anatomic changes and the organ motions during the course of treatment. Barker JL Jr. et al. reported that relative median loss in gross tumor volume was 69.5% and measurable anatomic changes were found throughout the fractionated radiotherapy in head-and-neck patients[4]. As a result of these changes the actual delivered dose deviates from the original planned dose distribution, potentially affecting the tumor control and the normal tissue complication rates.

Cone-beam computed tomography (CBCT) systems mounted on the linear accelerator has become available for image-guided radiotherapy (IGRT). Currently, there are two types of commercially available CBCT imaging systems: (1) the kV-CBCT system, which includes the Varian On-Board-Imaging (OBI) (Varian Medical Systems, Palo Alto, CA) and the Elekta XVI Synergy system (Elekta, Stockholm, Sweden); and (2) the Siemens MVision system (Siemens Medical Solutions, Malvern, PA) [5-7]. In our hospital, we commissioned an Elekta Synergy™ accelerator with on-board kV-CBCT in 2006. The main clinical application of CBCT is to improve the geometric accuracy of target localization in radiation therapy, where the volumetric images of patient acquired immediately before the treatment are registered to the reference planning CT images to correct the patient setup error [8,9]. KV-CBCT imaging has shown enough soft tissue contrast and spatial resolution for soft-tissue based setup, but the image quality is affected by the acquisition parameters. In principle the kV-CBCT data set can be used to calculate the dose distribution, which means that the planned dose distribution can be evaluated and verified on every treatment day [10]. In order to use CBCT images for dose calculation, the image pixel values need to be converted from dimensionless CT numbers to either electron or physical density. Methods for calibrating conventional fan-beam CT to electron density have been widely used in clinical dose calculation[11]. However, compared to conventional CT scanners, kV-CBCT images have increased artifacts and reduced contrast due to photon scatter. As a result, the calibration of kV-CBCT images for dose calculation is an active area of research [12-15].

The purpose of this study is to assess the feasibility of using a mapping method to calibrate the kV-CBCT images for dose calculation in head-and-neck and definitive brain tumor radiation treatments. By monitoring the dose that patient receives from each fraction, physicians will be able to track the dose distribution during the course of radiation therapy and modify the treatment plan as needed based on the actual dose delivered.

## Methods

### KV CBCT data acquisition

The kV CBCT images were acquired on a linear accelerator equipped with an integrated kV X-ray volumetric imaging system (Elekta, Synergy S, XVI, Crawley, UK). For imaging the head-and-neck and brain tumor patients, we used the following parameters: 100 kVp, S20 collimator and F0 filter, total 65 mAs and a high-resolution reconstruction (512 × 512). A total of about 650 projections were acquired in a full rotation. The CBCT images were reconstructed with slice thickness of 2.5 mm and then transferred to the treatment planning system (TPS, Philips Pinnacle3 V8.0d, Fitchburg, WI, USA) for image registration and dose calculations.

### KV-CBCT Stability

A phantom, Catphan-600 module CTP503 (Phantom Laboratory, NY) was used to evaluate the stability and uniformity of the CBCT numbers. The phantom has seven embedded rods made of different materials: air, PMP, LDPE, polystyrene, acrylic, Delrin and Teflon. Their electron densities relative to water range from 0.00 to 2.16. The CBCT image of the phantom was acquired every month for three consecutive months, and the CBCT numbers were obtained from the TPS and the relative electron densities were recorded accordingly. We also evaluated the maximal fluctuation in CBCT numbers on the image uniformity module part of the phantom.

### Calibration of relative electron density

For the dose calculation in a treatment planning system, the relative electron density or physical density of each voxel of the CT images is required for inhomogeneity corrections [11]. In this study, calibration of conventional CT (AcQsim CT Simulator, Philips Medical System, Cleveland, OH) number to physical density was performed on a CT phantom (CIRS model 062, Norfolk, VA). However, each individual patient's CBCT scan has a different scatter component that affects the HU measured in the image. A significant dose error was observed if we directly applied the calibration method for conventional CT to kV-CBCT.

We used a Region-of-interest (ROI) CT number mapping method similar to Richter's report to generate the CT number to physical density conversion curve for the dose calculation [14]. This process was applied to a single patient with head and neck cancer. The CBCT images for this patient were acquired at the same day of planning CT, so that there was no visible change in patient anatomy between the two images. A brief description of the calibration progress is as follows: (1) register the planning CT images and kV-CBCT images in the ADAC Pinnacle treatment planning system; (2) map the regions of interests (ROIs) from conventional CT dataset to the CBCT dataset, and record the mean CBCT number values of these ROIs, and (3) Generate the kV-CBCT numbers to physical density calibration curve based on the density values measured on the conventional CT.

### Clinical Implementation

Three head-and-neck cases and three brain patients with different tumor sites treated on Elekta Synergy were selected for retrospective evaluation of the accuracy of CBCT-based dose calculations. The head-and-neck cases included two natural killer/T-cell (NK/T) lymphoma cases and one nasopharyngeal carcinoma (NPC) case. For all patients, conventional CT was acquired with slice thickness of 5 mm and the target and critical structures were delineated by the attending physicians. IMRT plans were designed according to the physician's prescriptions with beams of 6 MV. The beam angles were 0, 50, 110, 250 and 310 degree for NK/T cases, and 0, 45, 90, 120, 160, 200, 240, 280 and 320 degree for NPC cases. For brain cases, gantry angles were 0, 60, 230, 300 and 45 degree with a 90-degree couch kick. Two sets of CBCT images were acquired, one on the first treatment day and the other on two weeks later. The patients were initially set up to the skin markers then followed with a CBCT scanning. The CBCT images were acquired according to the appropriate protocol and then reconstructed with slice thickness of 2.5 mm. All images were transferred to treatment planning system for analysis. For each case, the CBCT images were first registered to the conventional kV-CT images using an automatic registration method based on normalized mutual information algorithm, and then manual adjustments were performed to achieve the optimal match. A second set of CBCT images were acquired two weeks later and registered to the reference planning CT with the same method. The contours were mapped from CT to CBCT images with slight changes if them were beyond the skin.

For dose calculation, all the parameters (e.g., iso-center location, beam angles, MLC shapes, and monitor units) in the initial treatment plan were applied to the kV-CBCT images, and then the dose distribution was recalculated based on the new calibration curve. The dose calculation was performed in the Pinnacle treatment planning system using the collapsed cone superposition convolution algorithm with an isotropic 2 mm dose grid resolution. The contours delineated on the conventional CT were also mapped onto the kV-CBCT image data sets. Finally, the initial dose distribution matrix calculated on the planning CT was imported in the treatment planning system and displayed on the kV-CBCT dataset using scripts developed in-house. Dose volume histogram and the dose to tumor and normal structures were compared on the two image data sets. The differences in the dose distributions of the two plans were analyzed using γ analysis along planes through the isocenter perpendicular to each beam axis using commercial software (MapCheck, Version4.0, Sun Nuclear, Melbourne, FL)[16].

## Results

### The stability of kV-CBCT numbers

Because most patients complete their treatment courses within five weeks, we consider the three-month length of the stability test to be adequate. The maximal difference in CBCT numbers was 21, with a maximum standard error of less than 1.5%. The stability of kV-CBCT number and electron density indicates acceptable overall performance of the kV-CBCT system. The kV-CBCT images of the uniformity section of the phantom shows the maximal fluctuation of the CBCT numbers is ± 35 Hounsfield unit (HU), which translates to a fluctuation of approximately 1% in electron density values.

### Conversion of kV-CBCT numbers to relative electron density

**Figure 1:**
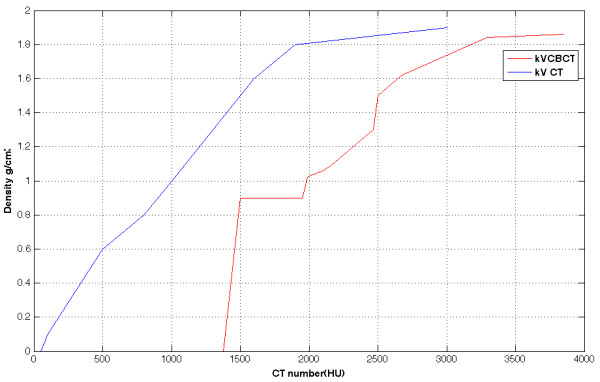
**Calibration curves for kV CT and kV CBCT**. The calibration curves for kV-CT and kV-CBCT based dose calculations in the treatment planning system.

A total of 13 different ROIs were used in generating the conversion curve, which include air, skin, muscle, brain stem, spinal cord, parotid gland, outer bone, inner bone, tooth and other structures. Table 1 shows the CBCT numbers and their corresponding physical density values. The calibration curves, as shown in Figure 1, were implemented in treatment planning system for the dose calculations. Large discrepancies were noted from these two curves. In particular, some discontinuous steps were observed on the calibration curve of kV-CBCT images.

**Table 1:** The densities and CBCT numbers.

Region of interest (ROI)	1	2	3	4	5	6	7	8	9	10	11	12	13
CBCT Numbers (HU)	0	1379	1500	1950	1990	2000	2103	2158	2468	2500	2670	3293	3847
Density (g/cm^3^)	0.0	0.0	0.9	0.9	1.02	1.03	1.06	1.09	1.30	1.50	1.62	1.84	1.86

The densities and CBCT numbers for generating the CBCT calibration curve

### Clinical cases

**Figure 2:**
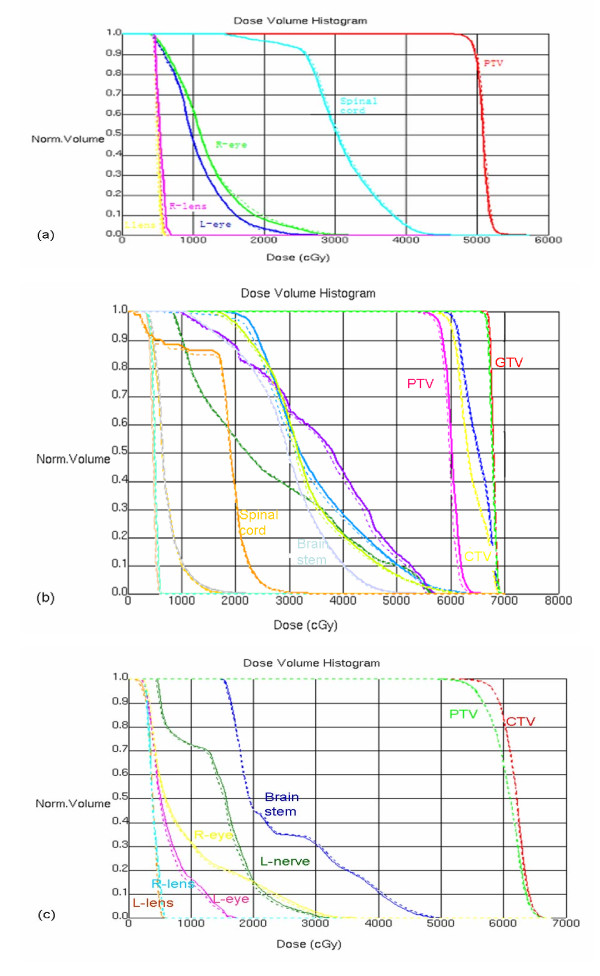
**DVH displays of three clinical cases**. The DVHs of three cases: one NK/T lymphoma (a), one NPC (b) and one Brain (c). The solid lines represent the dose based on conventional CT and the dash lines represent the dose based on kV CBCT.

**Figure 3:**
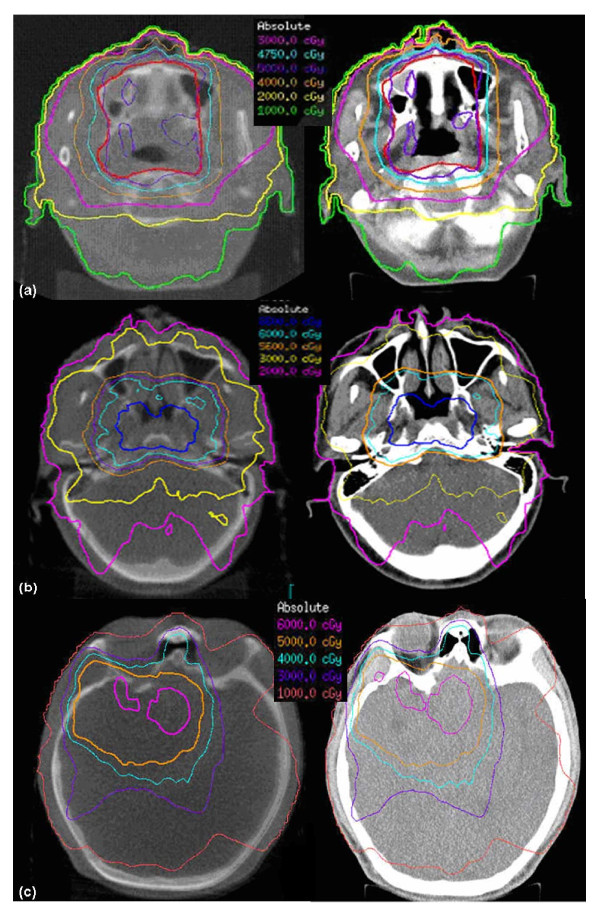
**Dose distributions of three clinical cases**. The transverse views of dose distributions of the NK/T lymphoma (a), NPC (b) and brain tumor (c). Left: calculated dose based on CBCT images; Right: calculated dose based on planning CT images. They show good agreement on both relative high and low isodoses.

Only minimal changes and deformations were observed in the anatomical structures on the patients' first CBCT images as compared with the reference planning CT. The DVHs of one NK/T lymphoma case (patient1), one NPC case (patient2) and one brain tumor case (patient3) are shown in Figure 2 as an example. The solid lines represented the DVHs based on conventional CT images and the dash lines were based on the dose calculated from the KV-CBCT images. Figure 3(a)-(c) are the dose distributions on the transverse planes of the three patients. The left images represent dose distributions based on the kV-CBCT and the right had images represent the dose on kV-CT images. There is no significant dose difference between the conventional CT images and kV-CBCT images.

For all clinical cases, the dose comparison was performed at a plane through the isocenter for each individual beam. Good agreement was found between the conventional CT and the first kV-CBCT based dose calculations. Using the γ analysis with a criterion of 2 mm and 2% and a threshold of 10%, more than 99.5% of the points at the iso-plane have the γ value less than 1.0. Table 2 shows the distance to agreement (DTA) and gamma analysis results of the three cases. For most of the beams, the pass rate for distance to agreement were better than 96% except one beam which has the data of 94.5%.

**Table 2:** The comparison of iso-plane dose distributions.

Patients	Beam index	DTA (3 mm, 3%, 10%) Pass rate (%)	Gamma index analysis (2 mm, 2%, 10%, γ<1) Pass rate (%)
NK/T	1	99.0	100.0
	2	100.0	100.0
	3	99.4	100.0
	4	99.5	100.0
	5	99.2	100.0
NPC	1	97.2	99.5
	2	98.8	99.8
	3	99.8	99.8
	4	98.9	100.0
	5	96.3	99.6
	6	94.5	99.9
	7	99.8	100.0
	8	99.5	100.0
	9	99.0	100.0
Brain Tumor	1	99.7	100.0
	2	99.8	100.0
	3	96.3	100.0
	4	100.0	100.0
	5	100.0	100.0

The comparison of iso-plane dose distributions based on conventional CT and KVCBCT for 3 clinical cases using distance to agreement (DTA) and gamma index analysis in all the beams.

Table 3 shows the dose to the tumor and some normal structures of the three patients in the planning CT data sets and the first CBCT data sets. The differences of the dose to tumor and some normal tissues were within 1% and 3.2%, respectively. The difference of maximal dose in tumor is 0.49% and in normal structures are 3.15%.

**Table 3:** Dose comparisons in targets and normal tissues.

Patients		1^st ^kVCBCT (Gy)	kVCT (Gy)	Difference (%)
NK-T lymphoma (patient1)	1PTV: mean dose	51.03	51.28	0.49%
	1Right-eye: mean dose	11.68	11.76	0.70%
	1Left-eye: mean dose	10.54	10.58	0.37%
	1Spinal cord: max dose	47.14	47.65	1.07%
	1Whole body: max dose	59.18	59.44	-0.43%
NPC (patient2)	2GTV: mean dose	67.8	67.78	-0.03%
	2CTV: mean dose	64.9	64.89	-0.02%
	2PTV: mean dose	63.76	63.78	0.03%
	2Brain Stem: max dose	53.83	54.36	0.98%
	2Spinal cord: max dose	32.85	32.73	-0.34%
	2L-parotid: mean dose	34.92	34.11	-2.37%
	2R-parotid: mean dose	33.38	32.91	-1.41%
	2Whole body: max dose	70.73	70.12	0.87%
Brain Tumor (patient3)	3CTV: mean dose	61.85	61.84	-0.02%
	3PTV: mean dose	60.64	60.57	-0.11%
	3Brain: max dose	50.13	50.02	-0.22%
	3Left-eye: mean dose	19.42	19.4	-0.13%
	3Right-eye: mean dose	9.94	9.64	-3.15%
	3Whole body: max dose	67.21	67.66	0.66%

Dose comparisons of the first kVCBCT to the planning kVCT in targets and normal tissues for the three clinical cases (NK-T lymphoma, NPC and Brain Tumor). The difference of maximal dose in tumor is 0.49% and in normal structures are -3.15%. The numbers before the contours are the indexes of patients, 1PTV means the PTV in patient1.

**Figure 4:**
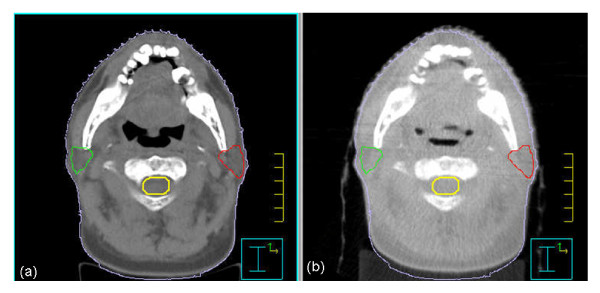
**The transverse views of CT and the 2nd CBCT**. The transverse views of the reference CT (a) and cone beam CT (b) after two weeks of the treatment. A slight change happened in the external contour and air cavity.

Five out of six patients didn't show significant anatomy changes and setup variations between the first CBCT images and the second CBCT images. But for one NK/T patient (patient5), a slight anatomical change in the patient's skin contour and air cavity was found in the second CBCT images compared to the conventional CT images, as shown in Figure 4. The dose comparisons of the reference kVCT, the 1st and 2nd kV CBCTs for that patient are listed in Table 4. On the first treatment day, the dose difference in gross tumor volume (GTV), clinical tumor volume (CTV) and planning tumor volume (PTV) between reference CT and cone beam CT (kV-CBCT1) were 0.98%, 0.54%, 0.54%, respectively. The maximal dose difference was found on the spinal cord (-1.87%). For the second cone beam CT (kV-CBCT2) acquired two weeks later, the maximal dose difference of spinal cord increased to 3.77%, and the maximal dose difference was found in the right parotid (5.81%). While for tumor and other structures, the dose agreement was still within 1.0%.

**Table 4:** Dose comparisons of the first and second CBCT images.

	Mean dose (Gy)					Maximal dose (Gy)		
	
	GTV	CTV	PTV	Left-parotid	Right-parotid	Brain-stem	Spinal-cord	Whole-body
kV CT	50.72	51.04	51.02	32.14	30.40	50.92	31.76	55.78
1^st ^kV CBCT	50.23	50.77	50.74	31.88	30.57	51.21	31.17	55.63
Dose difference between 1^st ^kV CBCT and kV CT(%)	0.98%	0.54%	0.54%	0.8%	-0.56%	0.57%	-1.87%	-0.26%
2^nd ^kV CBCT	50.37	50.77	50.76	32.56	28.73	51.36	32.96	55.71
Dose difference between 2^nd ^kV CBCT and kV CT(%)	0.7%	0.53%	0.52%	-1.3%	5.81%	0.86%	3.77%	-0.11%

Dose comparison of the first and second CBCTs to the reference planning kVCT in a patient (patient 4) with marked anatomical changes. The largest changes in dose difference between CBCT and planning CT are found in the spinal cord (from -1.87% to 3.77%) and in the right parotid (from -0.56% to 5.81%).

## Discussion

On-board CBCT volumetric imaging can improve the accuracy of radiation therapy in two aspects, namely target localization and delivered dose verification [12,17,18]. By acquiring 3D CBCT images with patient on the treatment couch just before the treatment delivery, patient setup error can be corrected and the accuracy of target positioning localization accuracy can be improved. CBCT image data sets obtained throughout the treatment course can be used for dose calculation, hence providing a clinical quality assurance tool for radiotherapy. However, the CBCT image quality is susceptible to many factors, such as scattering, beam hardening effects and organ motion, etc [19]. Morin et al studied dose calculation based on MV CBCT images and reported that the MV CBCT could be used to estimate the dose variation due to the anatomical changes in the head-and-neck region [20]. In this study, we investigated the feasibility and the accuracy of using kV CBCT images for direct dose calculation in head-and-neck and brain tumor radiotherapy with a simple and effective method.

The relative electron density can directly affect the dose calculation accuracy when inhomogeneity correction is involved. Unlike conventional kV CT or MV CBCT, kV-CBCT has a larger scatter radiation component and the image quality suffers from the beam hardening effect [21-23]. It has been reported that the effect of scatter radiation can be partly corrected or reduced by calibrating the kV-CBCT system [24,25]. The conventional CT number to the relative electron density conversion was performed with a CT number calibration phantom embedded with different types of tissue-equivalent inserts. However if such method was used directly for kV-CBCT, a dose calculation error can be introduced. Based on our tests, if we used the calibration curve generated by the phantom directly, the dose difference between first CBCT and planning CT would be more than 5%, which agrees with the reports from Yang et al and Tucking et al [12,26].

In this study, we used the ROI mapping method to generate calibration curve for kV CBCT image-based dose calculation [14]. Obviously, accurate image registration is needed for this method. The registration of different image modalities is widely used in radiotherapy for delineating the region of interests[27]. As the registration algorithms in the commercial treatment planning systems generally use rigid body transformations, we selected the head-and-neck and brain tumor cases for our study, where this assumption was generally valid.

The calibration curve for kV-CBCT is different from that for conventional CT. The conventional CT number is zero for the air outside the patient skin in the planning system; however, the CBCT number in such situation is much greater than zero. The mean CBCT value in the air around the skin is 1379 for the selected case, similar to other report [14]. The steps in the kV CBCT electron density conversion curve is mainly caused by scatter and beam hardening effects.

A good agreement of the calculated doses to the tumor and normal structures was found between the conventional CT and the first kV-CBCT images because there were virtually no anatomical changes between these images. The maximal dose deviation was found in the eye mainly due to the residual registration error and contour deviations, as the slice thickness was 5 mm for the conventional CT and 2.5 mm for CBCT images. The structures near the skin showed larger differences. The DTA and γ index analysis results also showed the good agreement between kV-CBCT based and conventional CT based dose calculation. Richter et al. used the same method and reported the dose difference between the planning CT and CBCT was 1.36% ± 1.96% in head patients with three-dimensional conformal plans. Our data showed the difference was within 1% of the target, which was consistent with their result. Our results demonstrated that the mapping method for CBCT correction is accurate both for three-dimensional conformal plans and IMRT plans in head and brain cases.

Furthermore, we generated the density conversion table based on one patient and applied the same table to the other patients. There were only a small discrepancy between the doses calculated by using kV CBCT and conventional CT in all 6 cases with different tumor locations. This result suggests that, for head-and-neck and brain patients, variations in the scatter effect in imaging different tumor sites is relatively small from patient to patient, and therefore it is reasonable to use the same electron density conversion curve for kV CBCT based dose calculation. Compared to the patient group based conversion table in report of Richter et al. or CT-based HU mapping method in Mathilda et al., this specific case mapping method is less complex to develop and implement, but it is limited to the preset scanning parameters.

Overall, our study showed good accuracy in CBCT based dose calculation. However, it is not recommended to replace the conventional planning CT by kV CBCT for the purpose of treatment planning as the inferior image quality of kV CBCT may affect the accuracy of target and normal structures delineation.

The kV CBCT can also be used to evaluate the dose to tumor or the normal structures. In this study, one NK/T patient had slight changes in anatomy after two weeks' treatment, dose variations were found in the spinal cord and the right parotid gland. These results suggested that even within a relatively short period such as 2 weeks, dose verification based on CBCT or CT will be necessary for certain patients to account for dosimetric effects due to patient anatomical changes.

Anatomic changes for head-and-neck patients, including nodal mass shrinkage and patient weight loss during the course of radiation therapy, can occur [28,29]. For these cases, repeat CT imaging and re-planning may be essential to ensure the adequate dose delivered to the tumor and proper sparing of the surrounding sensitive structures.

Technically, the 26 cm field of view for the S20 collimator may limit the use of kV CBCT for dose calculation of patients with beams going through their shoulders. However for most head-and-neck patients, the FOV is sufficiently large to evaluate the dose to PTV, brain stem, spinal cord, eyes and parotid glands. For those patients who receive thoracic or pelvic treatment, S20 is not large enough to encompass all the structures and skin. Dose verification for other sites is part of our future research.

## Conclusions

ROI mapping method is a feasible method to overcome the effects of scatter for generating the kV CBCT relative electron density calibration curve for head-and-neck cancer and brain tumor patients. Dose variations as monitored using kV CBCT imaging were observed in a relatively short period of two weeks, which suggests potential benefits of adaptive treatment plan re-optimization for certain head-and-neck and brain tumor patients.

## Declaration of competing interests

The authors declare that they have no competing interests.

## Authors' contributions

Each author has participated sufficiently in the work to take public responsibility for appropriate portions of the content. JY, ZZ designed the study. WH, JW performed the study and analysis. XM provided the patients' images. The manuscript was written by WH, all other authors helped and finally approved the final manuscript.
